# Squamous cell carcinoma of the ampulla of Vater: a rare case report and review of the literature

**DOI:** 10.1097/RC9.0000000000000628

**Published:** 2026-07-02

**Authors:** A. Dafnis, A.V. Rompou, M. Gazalidou, A. Leventi, D. P. Korkolis

**Affiliations:** a Department of Surgical Oncology, Agios Savvas Anticancer Hospital, Athens, Greece; b Department of Pathology, Agios Savvas Anticancer Hospital, Athens, Greece

**Keywords:** ampulla of Vater, pancreaticoduodenectomy, periampullary cancer, rare tumor, squamous cell carcinoma

## Abstract

**Introduction:**

Primary squamous cell carcinoma (SCC) of the ampulla of Vater is an exceptionally rare malignancy, with only a limited number of cases reported in the literature. Its pathogenesis, biological behavior, and optimal management remain poorly defined.

**Case presentation:**

We report the case of a 68-year-old man presenting with several weeks of atypical abdominal discomfort and cholestatic liver enzyme abnormalities. Upper gastrointestinal endoscopy revealed an ulcerated ampullary lesion, and biopsy confirmed moderately to poorly differentiated SCC. Cross-sectional imaging excluded metastatic disease. The patient underwent pylorus-preserving pancreaticoduodenectomy. Histopathological analysis confirmed primary SCC (pT2N0M0) with negative margins (R0 resection). Adjuvant chemotherapy with cisplatin and 5-fluorouracil was initiated 6 weeks postoperatively, with a planned duration of 6 months. The patient remains disease-free at 6-month follow-up.

**Discussion:**

Ampullary SCC is rare but not unique, with several cases reported. Surgical resection remains the cornerstone of treatment. Adjuvant therapy is extrapolated from other SCCs due to a lack of evidence. Prognosis appears variable, with better outcomes in node-negative disease.

**Conclusion:**

This case highlights the importance of accurate histopathological diagnosis and reinforces the role of radical surgical resection. Further case accumulation is required to define optimal management strategies.

## Introduction

Neoplasms of the ampulla of Vater account for less than 1% of gastrointestinal malignancies^[^1^]^. While adenocarcinoma constitutes more than 90% of cases, primary squamous cell carcinoma (SCC) is extremely rare. Although uncommon, several cases have been reported in the literature, indicating that this entity is rare but not unique^[^1–10^]^.

The ampulla represents a complex anatomical junction of biliary, pancreatic, and intestinal epithelium, making the occurrence of pure SCC biologically unusual. Proposed mechanisms include squamous metaplasia secondary to chronic inflammation, malignant transformation of pluripotent epithelial cells, or squamous overgrowth of an undifferentiated carcinoma^[^1,3,8,11^]^.

Patients typically present similarly to ampullary adenocarcinoma, often with obstructive jaundice. However, atypical presentations may occur, contributing to diagnostic delays. Definitive diagnosis frequently relies on postoperative histopathological analysis.

HIGHLIGHTS

- Primary squamous cell carcinoma of the ampulla of Vater is exceptionally rare.
- Definitive diagnosis requires the exclusion of adenosquamous and metastatic disease.
- Pancreaticoduodenectomy remains the mainstay of curative treatment.
- Histopathology and immunohistochemistry are essential for accurate diagnosis.
- Close postoperative surveillance is warranted due to potentially aggressive behavior

This case is reported in accordance with the SCARE 2025 guidelines^[^12^]^.

## Presentation of case

A 68-year-old man with a history of hypertension and dyslipidemia, treated with amlodipine and atorvastatin, respectively, presented with several weeks of nonspecific abdominal discomfort. He was a non-smoker, reported occasional alcohol consumption, and had no relevant family history of malignancy.

Physical examination was unremarkable. Laboratory investigations demonstrated a cholestatic pattern (ALP 243 U/L, γ-GT 229 U/L), with normal bilirubin, transaminases, and tumor markers (CA 19-9 and CEA).

Initial evaluation included transabdominal ultrasound and colonoscopy, both of which were unremarkable and did not identify a cause for the patient’s symptoms. Given the presence of a cholestatic biochemical profile and persistent clinical suspicion for periampullary pathology, upper gastrointestinal endoscopy (esophagogastroduodenoscopy) was subsequently performed to allow direct visualization of the ampullary region. This revealed a 2–3 cm ulcerated, friable lesion at the ampulla of Vater. Histological examination of biopsy samples confirmed moderately to poorly differentiated SCC.

**Figure 1.**
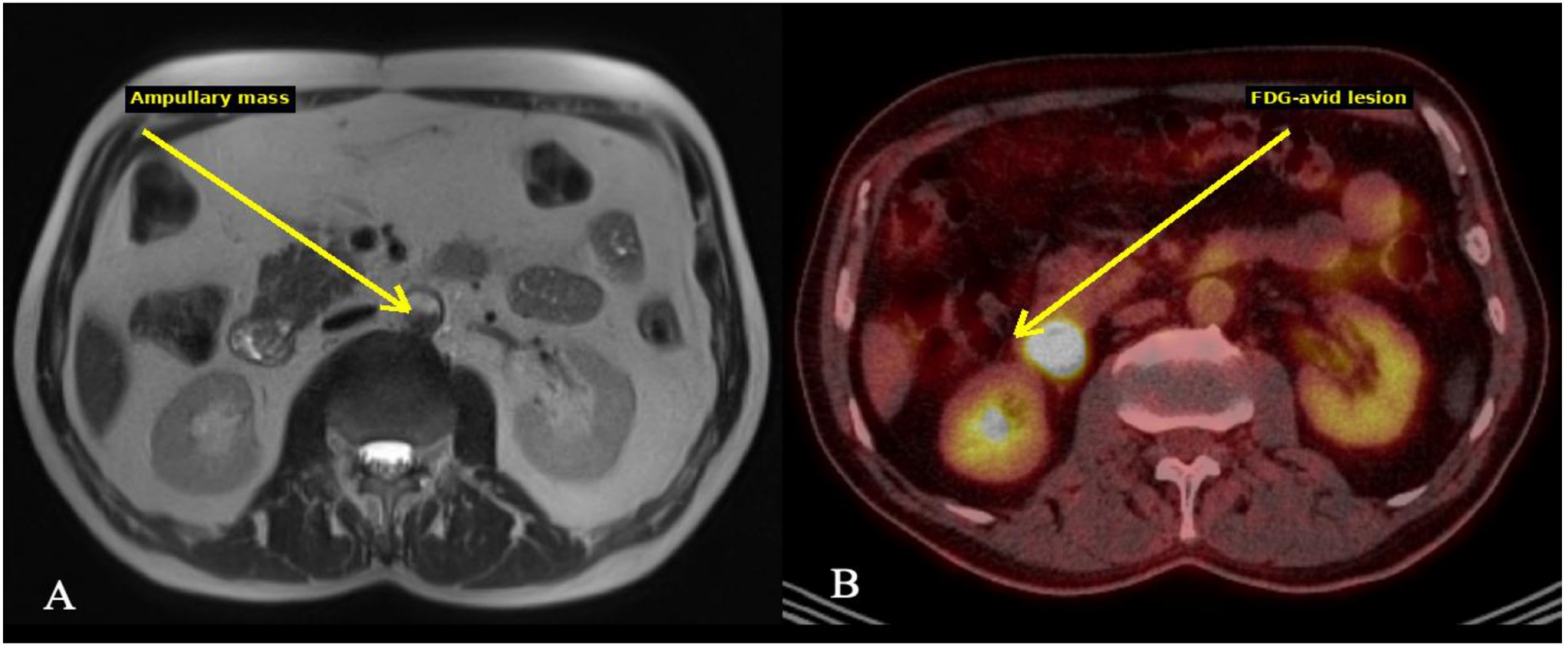
Pre-operative imaging. (A) Axial MRI demonstrating the ampullary mass (arrow) at the level of the pancreatic head, with associated biliary ductal changes. (B) PET-CT showing a focal FDG-avid lesion (arrow) at the ampulla of Vater, with no evidence of nodal or distant metastatic disease.

Subsequent contrast-enhanced computed tomography (CT), magnetic resonance imaging (MRI)/magnetic resonance cholangiopancreatography, and positron emission tomography (PET)-CT demonstrated a localized ampullary mass without nodal or distant metastases (Fig. 1A and 1B).

Following a multidisciplinary discussion, the patient underwent an elective pylorus-preserving pancreaticoduodenectomy. This approach was selected due to the absence of gastric or proximal duodenal involvement, allowing for the preservation of gastric emptying without compromising oncological clearance. The procedure was performed by a senior hepatopancreatobiliary surgeon.

Intraoperatively, a localized ampullary mass was identified without evidence of metastatic spread. The postoperative course was uneventful. The patient received piperacillin–tazobactam for 5 days, thromboprophylaxis, and early enteral nutrition. No complications were observed, and he was discharged on postoperative day 10.

**Figure 2.**
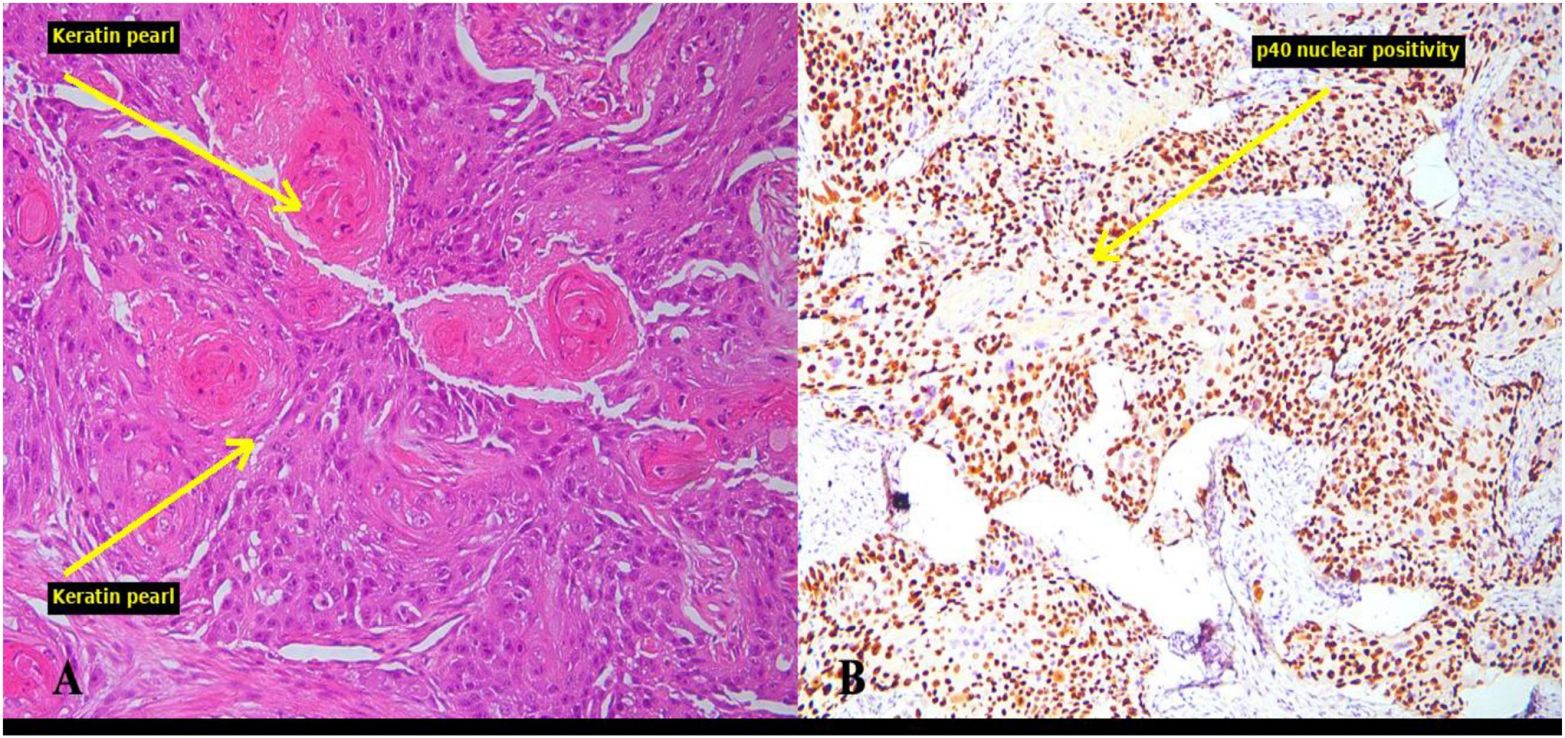
Histopathological findings. (A) Hematoxylin and eosin staining demonstrates moderately to poorly differentiated squamous cell carcinoma with characteristic keratin pearls (arrows), confirming squamous differentiation. (B) Immunohistochemistry shows diffuse strong nuclear p40 positivity (arrow), supporting the diagnosis of primary squamous cell carcinoma.

Histopathological examination revealed a 3.2 cm moderately to poorly differentiated SCC with keratinization (Fig. 2A and 2B). Immunohistochemistry showed positivity for p40 and CK5. No glandular component was identified. Thirty-one lymph nodes were negative, confirming pT2N0M0 disease. Resection margins were clear (R0).

Adjuvant chemotherapy with cisplatin and 5-fluorouracil was initiated 6 weeks postoperatively, with a planned duration of 6 months.

At 6-month follow-up, the patient remains disease-free. The clinical course from symptom onset to 6-month follow-up is summarized in Table 1.

**Table 1:** Timeline of events.

Event	Timepoint
Symptom onset	Several weeks prior
Initial assessment	Day 0
EGD and biopsy	Week 1
Imaging (CT/MRI/PET)	Week 2
MDT decision	Week 3
Surgery	Week 4
Discharge	Postoperative day 10
Chemotherapy initiation	Week 10
Follow-up	6 months

EGD, esophagogastroduodenoscopy; MDT, multidisciplinary team.

## Discussion

Primary SCC of the ampulla of Vater is a rare malignancy, with only a small number of reported cases^[^1–10^]^. Despite its rarity, it is increasingly recognized as a distinct pathological entity.

Accurate diagnosis requires the exclusion of adenosquamous carcinoma and metastatic SCC from other primary sites, as secondary involvement of the ampulla by squamous carcinomas of the larynx or esophagus has been documented^[^13,14^]^. Immunohistochemistry plays a key role in confirming squamous differentiation.

Compared with previously reported cases, our patient demonstrated favorable features, including node-negative disease and complete resection. Literature suggests that these factors are associated with improved survival, with some patients surviving beyond 2 years^[^6,14^]^. A summary of previously reported cases of primary SCC of the ampulla of Vater, including surgical approach, adjuvant treatment, and outcomes, is presented in Table 2.

**Table 2:** Summary of previously reported cases of primary squamous cell carcinoma of the ampulla of Vater, including age/sex, clinical presentation, pathological stage, surgical procedure, adjuvant therapy, and outcome.

Author (Year)	Age/Sex	Presentation	Pathological stage	Surgical procedure	Adjuvant therapy	Outcome/Survival
Puderbach & Bellamy (1952)^[^5^]^	N/R	Obstructive jaundice	N/R	Surgical resection	None reported	First reported case; outcome not reported
Marin-Padilla & Dewan (1960)^[^6^]^	N/R	Obstructive jaundice	N/R	Surgical resection	None reported	Survival beyond 2 years reported
Chen *et al* (1996)^[^4^]^	72/F	Obstructive jaundice, biliary infection	N/R	PTCD drainage (unresectable)	Palliative	Poor; died of disease
Deepak & Mishra (2008)^[^7^]^	30/F	Jaundice, abdominal pain, anorexia	N/R (node-negative)	Classic Whipple’s (PD)	None reported	R0 resection achieved; follow-up not reported
Bolanaki *et al* (2014)^[^3^]^	68/M	Painless obstructive jaundice, fatigue, weight loss	pT3N0M0 (Stage IIA); R0	Classic Whipple’s (PD)	None reported	Liver metastases at 4 months; died at 5 months post-op
Balci *et al* (2016)^[^2^]^	54/F	Weight loss, jaundice, epigastric pain	N/R	Classic Whipple’s (PD)	Adjuvant chemotherapy recommended	Uneventful postoperative course; long-term outcome not reported
Reddy *et al* (2016)^[^9^]^	N/R	Obstructive jaundice	N/R	Surgery + chemoradiotherapy	Chemoradiotherapy (multimodality)	Disease-free at reported follow-up
Sekhri *et al* (2018)^[^10^]^	N/R	Obstructive jaundice	N/R	PD (Whipple’s)	N/R	Outcome not reported
Soni *et al* (2021)^[^1^]^	N/R	Obstructive jaundice	N/R	PD with curative intent	Adjuvant chemotherapy	Disease-free at reported follow-up
Present case (2025)	68/M	Atypical lower abdominal discomfort; cholestatic enzymes	pT2N0M0; R0	PPPD (pylorus-preserving)	Cisplatin + 5-FU (6 months)	Disease-free at 6 months (follow-up ongoing)

N/R, not reported; PD, pancreaticoduodenectomy; PPPD, pylorus-preserving pancreaticoduodenectomy; PTCD, percutaneous transhepatic cholangio-drainage; R0, microscopically margin-negative resection; 5-FU, 5-fluorouracil.

Surgical resection remains the cornerstone of treatment. Pancreaticoduodenectomy, including pylorus-preserving variants, offers the best chance of cure^[^1–4,7–9^]^. The choice of pylorus preservation in this case was based on tumor location and absence of gastric involvement, consistent with established oncological principles for periampullary malignancies^[^2,4,7^]^.

The role of adjuvant chemotherapy remains unclear. Platinum-based regimens, such as cisplatin combined with 5-fluorouracil, are commonly used by extrapolation from other SCCs, although evidence is limited^[^2,8,9^]^. Reported outcomes are heterogeneous, with variable responses to systemic therapy^[^1,8^]^.

Ampullary SCC may demonstrate more aggressive behavior compared to adenocarcinoma, with early recurrence reported in several cases^[^1,5,8^]^. However, long-term survival has been observed in selected patients, particularly those with node-negative disease and complete (R0) resection^[^6,14^]^.

Postoperative surveillance typically involves clinical review and imaging every 3–6 months during the first 2 years, reflecting patterns of recurrence observed in previously reported cases^[^7,10^]^.

A limitation of this report is the relatively short follow-up period of 6 months, which does not allow for the assessment of long-term outcomes. Given that recurrence in ampullary and other SCCs may occur within the first 2 years, continued surveillance is planned with clinical review and cross-sectional imaging every 3–6 months, and updated outcomes will be reported as follow-up matures^[^1,7^]^. Additionally, the absence of intraoperative and gross specimen photographic documentation limits the visual educational value of this case.

Further studies and case accumulation are required to better define prognosis and optimize treatment strategies.

## Conclusion

Primary SCC of the ampulla of Vater is an exceptionally rare malignancy. Radical surgical resection remains the mainstay of treatment. Given the lack of standardized management protocols, individualized multidisciplinary approaches are essential. Continued reporting of such cases will improve understanding and guide future therapeutic strategies.

## Ethical approval

This study is a single, retrospective, anonymised case report and did not require formal ethical approval under the policy of the Institutional Review Board of Agios Savvas Anticancer Hospital, Athens, Greece. Written informed consent was obtained from the patient for publication of this case report and the accompanying images.

## Data Availability

The data supporting the findings of this case report are available within the article. Any further enquiries can be directed to the corresponding author on reasonable request.

## References

[1] Soni S Elhence P Varshney VK. Primary squamous cell carcinoma of the ampulla of Vater: management and review of the literature. BMJ Case Rep 2021;14:e236477. 10.1136/bcr-2020-236477 PMC7797242 33414110

[2] Balci B Calik B Karadeniz T. Primary squamous cell carcinoma of the ampulla of Vater: a case report. Surg Case Rep 2016;2:2. 26943678 10.1186/s40792-016-0130-0 PMC4706539

[3] Bolanaki H Giatromanolaki A Sivridis E. Primary squamous cell carcinoma of the ampulla of Vater. JOP 2014;15:42–45. 24413783 10.6092/1590-8577/1649

[4] Chen CM Wu CS Tsai SL. Squamous cell carcinoma of the ampulla of Vater: a case report. Changgeng Yi Xue Za Zhi 1996;19:253–57. 8921644

[5] Puderbach WJ Bellamy J. Squamous-cell carcinoma of the ampulla of Vater. Surgery 1952;32:110–13. 14950595

[6] Marin-padilla M Dewan CH. Squamous-cell carcinoma of ampulla of Vater. Guthrie Clin Bull 1960;29:148–53. 13766768

[7] Deepak KS Mishra PK. Squamous cell carcinoma of the ampulla of Vater: a rare case successfully managed by pancreaticoduodenectomy. HPB (Oxford) 2008;10:378.

[8] Gupta A Kumar S Kumaresh TS. Primary squamous cell carcinoma of the ampulla of Vater: a rare entity. Internet J Surg. 2010;22.

[9] Reddy S Bangri SA Dinesh K. Multimodality therapy for primary squamous cell carcinoma of the ampulla of Vater: report of a rare case with literature review. JOP 2016;17:679–82.

[10] Sekhri R Kamboj M Gupta G. Primary squamous cell carcinoma of the ampulla. Indian J Pathol Microbiol 2018;61:300–02. 29676387 10.4103/IJPM.IJPM_340_17

[11] Pathak GS Deshmukh SD Yavalkar PA. Coexistent ampullary squamous cell carcinoma with adenocarcinoma of the pancreatic duct. Saudi J Gastroenterol 2011;17:411–13. 22064341 10.4103/1319-3767.87184 PMC3221117

[12] Kerwan A Al-jabir A Mathew G. Revised Surgical CAse REport (SCARE) guideline: an update for the age of Artificial Intelligence. Prem J Sci 2025;10:100079.

[13] Büyükçelik A Ensari A Sarioğlu M. Squamous cell carcinoma of the larynx metastasized to the ampulla of Vater. Tumori 2003;89:199–201. 12841672 10.1177/030089160308900219

[14] Sreenarasimhaiah J Hoang MP. Esophageal squamous cell carcinoma with metastasis to the ampulla. Gastrointest Endosc 2005;62:310–11. 16047006 10.1016/s0016-5107(05)00556-0

